# The Psychological Expectation of New Project Income Under the Influence of the Entrepreneur’s Sentiment From the Perspective of Information Asymmetry

**DOI:** 10.3389/fpsyg.2020.01416

**Published:** 2020-07-08

**Authors:** Huaqian Zhong, Runyu Yan, Shuai Li, Min Chen

**Affiliations:** ^1^School of Mechano-Electronic Engineering, Xidian University, Xi’an, China; ^2^School of Law, East China Normal University, Shanghai, China; ^3^School of Social Development, East China Normal University, Shanghai, China; ^4^School of Innovation and Entrepreneurship, Wenzhou Medical University, Wenzhou, China

**Keywords:** information asymmetry, entrepreneur sentiment, psychological expectation of returns, initial public offering initial return, initial public offering pricing mechanism

## Abstract

The phenomenon of high first-day income and high break-up rate in China’s capital market has long attracted the attention of investors. Based on the disagreement model, in combination with the information asymmetry theory and behavioral finance, a mathematical model was put forward to analyze the reasons and mechanisms for the excess return of the initial public offering (IPO) on the first-day under the influence of investors’ sentiments. The analysis shows that the IPO first-day income is a function of the disagreement between investors, which generally presents an asymmetric U shape. Information asymmetry affects the degree of valuation deviation. The sentiment of investors reflects the psychological state of the investor at the time, which makes the income increase or decrease under the influence of investor sentiment and market sentiment. In turn, it leads to the emergence of excess returns or break-up rates on the first-day. The research results help deepen the understanding of the IPO pricing mechanism and explore the impact of investor psychology on its pricing, putting forward suggestions for the issue pricing mechanism of the Sci-Tech Innovation Board.

## Introduction

In China, the stock market started as a pilot in 1989. The original intention of setting up the Chinese stock market was to serve the poverty alleviation of state-owned enterprises. Even though it has developed so far, the stock market still focuses on financing over returns (Gao et al., 2018). At present, the A-share (RMB common stock) market has formed a hierarchical market system for the main board, small- and medium-sized enterprise (SME) boards, and growth enterprise market (GEM) in China. The requirements for listing conditions and listing entities are different in each market. The primary market (issuance market) is the basis and prerequisite for the secondary market (circulation market) (Chen J. et al., 2018). Among them, the primary market refers to the securities issue market, that is, the market where the stocks are traded for the first time. Commonly, the initial public offering (IPO) and new share offering of the company belong to the primary market (Tripathi and Pandey, 2018). Accordingly, the establishment of the SME board can accumulate supervision and operation experience for the establishment of the GEM, and its ultimate purpose is to build a multilevel capital market (Chen and Wang, 2018; Kotlar et al., 2018; Funaoka and Nishimura, 2019).

The fluctuation of China’s capital market is relatively obvious, and the phenomenon of “rise and fall suddenly and sharply” is very serious. This sharp rise and fall have also led many scholars to question the authenticity of stock prices reflecting the value of stocks (Blankespoor et al., 2017). The cause of capital market fluctuations can never be quantitatively analyzed, and it is related to many factors such as investor sentiment, investment psychology, fundamentals, and policies. Whether it is an accurate grasp of the policy environment or the concerted action of shareholders, the most difficult thing for investors to overcome is the asymmetry of information.

As an important way for companies to raise funds directly from investors in the public stock market, IPO can enable listed companies to expand and develop while playing a role in connecting the financial market and the real economy (Widarjo and Bandi, 2018). However, in the process of IPO issuance, it is common for IPO underpricing and continuous limit-up after listing. If the IPO appears excess returns in the short term, it will greatly raise the overall stock price of the secondary market, making it deviate from the true value of the stock. Then, the effectiveness of resource allocation in the stock market cannot be brought into play (Thomadakis et al., 2016).

Aiming at this abnormal phenomenon that affects the healthy development of the stock market during the IPO issuance and operation, this paper considers the factor of investor sentiment. Based on the information asymmetry theory and behavioral finance viewpoint, the causes and mechanisms of IPO abnormal initial returns are further explained under the influence of investor sentiment. The purpose of this study is to gain a deeper understanding of the IPO pricing mechanism and to explore the influence of investor psychology on its pricing. It also puts forward some suggestions for the issue pricing mechanism of the Sci-Tech Innovation Board to alleviate the pressure in the stable development process after IPO.

## Literature Review

Information asymmetry refers specifically to the fact that the parties have different information in the transaction. In actual economic activities, some members have information that other members do not, resulting in differences in the understanding of relevant information by various types of personnel. Usually, the party with more comprehensive information is in a more advantageous position, while the party with poor information is in a disadvantageous position. George (1970) analyzed the effect of information asymmetry on market behavior earlier. He analyzed the effect on buyers due to the uncertainty of product quality from the aspects of used car market, insurance market, and credit market. In a market with asymmetric information, the theoretical equilibrium price will quickly aggregate all the information. Thus, a trader will infer all the information he needs from the observable stock price. It shows that the stock price at this time is the price under the separation equilibrium, that is, the change in the transaction price reflects the change in the information set. Under these information sets, their respective equilibrium prices are different (García-Sánchez and Noguera-Gámez, 2017).

Some scholars have used the two-step regression analysis framework of Avramov and Chordia to test the explanation of the conditional asset pricing model containing investor sentiment information on China’s stock market return anomalies (Chen C. et al., 2018). The results show that the conditional asset pricing model (CAPM), which contains information on investor sentiment as the IPO first-day yield, can significantly explain the phenomenon of idiosyncratic volatility in China’s stock market. Then, it confirms the important role of investor sentiment factors in the asset pricing process. Based on related investigations, the information asymmetry theory is integrated to make an in-depth analysis of the reasons and mechanism of the IPO’s first-day excess returns. With the deepening investigation on IPO underpricing, the research on IPO underpricing based on behavioral finance occupies a dominant position. Clarke et al. (2016) used India’s IPO system and data to test the sentiment-based IPO underpricing model. Through a sample survey of 362 new share issuances in India from 2003 to 2014, the average IPO underpricing rate is 23%. Then, the underpricing is decomposed into two parts: one part is related to the underwriters’ voluntary underpricing, and the other part is related to the IPO trading activities on the first-day. It is found that the IPO underpricing rate caused by trading activities on the first-day accounts for a higher proportion of the total underpricing (Clarke et al., 2016).

After combing the investigation status of the above scholars, it is found that scholars in this field have formed a certain theoretical system for IPO underpricing. Moreover, more investigations on IPO underpricing are conducted from the perspective of behavioral finance, while investigations on information uncertainty and investor heterogeneity expectations are scarce. The hot issue of listed companies’ IPO underpricing is taken as the research object. Through the collection and sorting of related literature and theories, the corresponding summary and analysis of the scholars’ views are made. From the two perspectives of investor sentiment and information uncertainty, its impact on IPO underpricing is jointly explored, and corresponding hypotheses are proposed.

## Materials and Methods

### Information Asymmetry in Market Transactions

During the IPO, it mainly involves market participants such as government regulators, various intermediaries, investors, and listed companies (Chen C. et al., 2018). Information asymmetry theory means that in orderly market transactions, there are differences in market participants’ ability to acquire market information and information holdings. In real economic transactions, due to the asymmetry of market information, different investors have varied perceptions of the two indicators of investment risk and expected return. Usually, the party with more effective market information is more accurate and reliable in decision-making to obtain more benefits (Clarke et al., 2016). Also, this phenomenon will make the disadvantaged information holders increase transaction costs in order to obtain more accurate information and gain more benefits in market transactions.

If the information asymmetry in the market occurs before multiple parties sign a transaction agreement, it is an adverse selection and it is reasonable. However, if information asymmetry still exists after the transaction agreement is signed, it is a moral hazard and a potential hidden danger to the stock market bubble (Nagar et al., 2019). Whether it is adverse selection or moral hazard, there are certain harms to both sides of the transaction. For listed companies that adopt IPO financing, based on the information asymmetry theory, the company’s management tends to avoid some real information that may damage its image when describing its business situation. It discloses positive information about the company to the outside. Also, this part of the information in its own right does not rule out exaggeration and fiction (Abdel-Rahim and Stevens, 2018; Balakrishnan et al., 2019; Crowley et al., 2019). In this environment, due to the relatively insufficient information acquisition ability and information holding capacity of the transaction object, companies are likely to make wrong decisions. In this way, listed companies will occupy a dominant position in financing activities. At the same time, however, long-term information asymmetry will cause investors to lose their investment confidence due to economic losses, which will hinder the financing plan of listed companies and lead to market stagnation (Wu et al., 2020). From this point of view, information asymmetry will bring risk game operation in the process of large investment, and investors need to raise the investment after weighing.

In real market transactions, both parties to the transaction have made it clear that investors in the securities market, as providers of funds required for the development of listed companies cannot obtain the same information as company management (Crome et al., 2017). Therefore, at this time, the listed company is in an obvious advantageous position. Its investor is restricted by incomplete information, and it is impossible to accurately judge the market risk and expected return of the company. Therefore, it is necessary to choose a more stable investment scheme after comprehensive consideration.

### The Influence of Investor Sentiment on the Market

Investor sentiment is one of the main theories of behavioral finance to explain market anomalies. From a behavioral finance perspective, investors with heterogeneous beliefs and preferences are often irrational. Psychological factors play a very important role in their decision-making and investment processes. The large fluctuations in investor sentiment will cause deviations in market perception, which will cause the stock price to be affected by investor sentiment to a certain extent to change trends. To analyze stock market investors from the perspective of psychology and behavioral finance, they all have some irrational psychology and behavior. This sentiment will further be invested in the stock market and spread and eventually lead to market pricing deviations, which will cause stock prices to deviate from the market’s true value (Osu et al., 2017; Madison et al., 2018).

**FIGURE 1:**
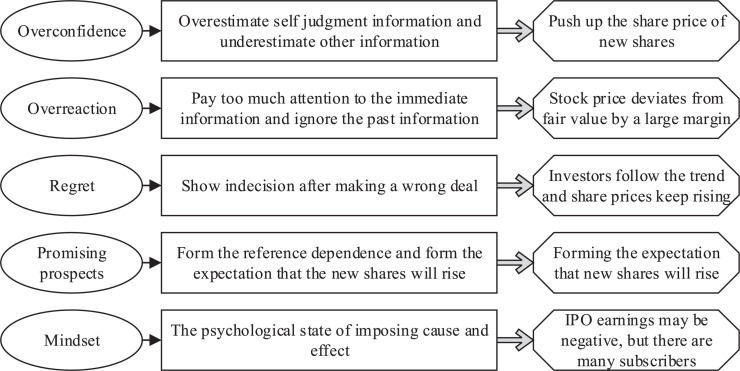
Five main manifestations and theories of investor sentiment.

As early as the mid-1990s, scholar Stein has proposed that a large part of investors’ judgments on future expectations come from investor sentiment. Investors’ wishes or expectations in the market can be directly reflected in investor sentiment. And this kind of non-material feedback cannot be quantified. Investors in the market can only subjectively understand the retention and change of sentiment. Investor sentiment is a very important concept in trading activities. The changing mood will directly affect investors’ judgment of expected returns, thereby changing their investment behavior (Woods et al., 2019). When investors receive market information, they will amplify the positive feedback of various positive and negative information. Investors generally have more market anomalies during periods of high sentiment than during periods of low sentiment. At present, many scholars have confirmed that changes in investor sentiment are important factors that cause many anomalies in the stock market. This paper believes that investor sentiment is an irrational judgment caused by a subjective preference of investors when they are in a positive or negative feeling. Investor sentiment can be divided into five manifestations. The related manifestations and theories are shown in Figure 1.

As can be seen from Figure 1, overconfidence means that individuals believe that their cognitive level is higher than others; that is, they will overestimate their own judgment and underestimate other information. Also, individuals will have self-attribution bias; that is, success is due to their ability, and failure is mostly attributed to external factors. Overreaction will strengthen investors’ optimism and make them ignore the previous experience that the skyrocketing will trigger a plunge. Lessons from history cannot prompt investors to become more rational. Investors only think more about short-term experience, but less about how far they deviate from the long-term average. The market is always overreacting. In the regret theory, it is believed that after an investor makes a mistake or wrong transaction, to avoid operating regret, he usually shows a mental state of indecision. Avoiding regrets usually leads investors to follow the crowd. If a large number of investors suffer losses due to the same operations as themselves, the sensitivity of investors’ psychological reactions will decrease. Promising prospect theory describes the behavior of individuals inconsistent with traditional expectation theory in risk decision-making. In the IPO market, since most of the new shares are rising, it will give investors a reference dependency, forming an expectation that the new shares will rise. The mindset is widespread in China’s IPO market. Since the underpricing rate in China’s IPO market has always been high, it is usually possible to obtain benefits only after winning the bid. Even if the IPO benefits may be negative at a certain stage, the number of applicants is still large.

### Theoretical Basis for Investor Disagreement

Disagreement refers to the different judgments that different investors have on returns under the same holding period of the same stock. This inconsistent view can also become a “heterogeneous belief.” Miller first synthetically considered disagreement and short selling obstacles in 1977. He believes that investor disagreement will lead to a decline in future stock returns (Coall et al., 2018). The greater the investor disagreement, the stronger the issuer’s restrictions on short selling of the stock, which means that the stock price is largely overvalued. When the stock price returns to normal levels, the future return of the stock will decrease accordingly. On this basis, some scholars further analyzed that due to the short-selling restrictions set by enterprises, the opinions of pessimistic investors were not reflected in the stock trading market. Once the optimistic investors exit the market, the role of the pessimistic investors will gradually turn into a marginal buyer, revealing a lot of previously hidden market information. When this situation occurs on a large scale, the market will collapse.

Comprehensive worldwide research can confirm that investor disagreement can predict the future returns of the stock market. Some scholars have derived a risk asset price equilibrium model based on the Miller theory. It is theoretically confirmed that the stock price not only is affected by the operation of the enterprise but also has a lot to do with the degree of disagreement among investors (Edeling and Himme, 2018; Zhang et al., 2018; Ademola et al., 2019). When investor disagreement is more obvious, the stock price is on the rise. If the degree of investor disagreement is low and the opinions are more unified, the stock price will decline. It also confirms Miller’s theory that disagreement can cause stock prices to be overvalued.

### Measure of Investor Sentiment

There are many ways to measure investor sentiment, and it is important to choose a suitable standard. The direct indicator is to collect the personal subjective feelings and opinions of investors on the market through questionnaire surveys. The key indicator is the investor confidence index. If the investor confidence index is greater than 50, it indicates an upward trend in the market; if the investor confidence index is less than 50, it means that the possibility of market volatility will increase. The confidence index can reflect the motivational tendency of investors to join the market (Amini and Toms, 2018; Härkänen et al., 2019). The indirect indicator is based on the ultimate market behavior of investors. Therefore, it can reflect investor sentiment more objectively than the direct indicator. Taken together, the direct and indirect indicators can reflect investor sentiment to some extent, but there are certain limitations. At present, more scholars are still working on related research on the measure of investor sentiment to more comprehensively reflect investor sentiment in market transactions (Chen, 2019).

Direct indicators are direct statistics on the sentiment of sentimental subjects (individual investors) through questionnaires and interviews. Investor confidence index and consumer confidence index are typical representatives of direct indicators. Indirect indicators are used to replace investor sentiment by selecting objective transaction data from some markets, such as the turnover rate and the number of new account openings. Indirect indicators are more objective and available, which makes them more analytically valuable. Thus, most of the relevant investigations focus on how to use indirect indicators to build an accurate and effective comprehensive investor sentiment indicator. The principal component analysis is used to construct a comprehensive investor sentiment indicator based on indirect indicators that may be related to investor sentiment.

### Empirical Research on the Influence of Investor Sentiment on Initial Public Offering Excess Returns

#### Variable Selection

Explanatory variables: investor sentiment indicator (market and individual stocks). The comprehensive return rate of the IPO market can directly or indirectly affect the investment behavior of investors. This paper selects the cumulative yield of the Shanghai stock index 1 day before issuance as a surrogate indicator of market sentiment to ensure the originality and validity of the data. This paper selects the three indicators of the winning rate, the oversubscribed multiple and the price–earnings ratio difference as indicators of investor sentiment related to individual stocks. Control variables: company size, profitability level, and listing board.

#### Model Construction

The summarized results of the variables are shown in Table 1. The descriptive statistical analysis results of each variable are shown in Table 2 (mean, standard deviation, maximum, and minimum).

**TABLE 1:** Variable selection.

Variable classes	Abbreviation	Full name	Explanation
Main explanatory variables	MR	Market investor sentiment	Cumulative yield of Shanghai stock index 1 day before issuance
	LOTTERY	Winning rate of issuance	Number of shares issued online/number of shares effectively subscribed online
	OVERSUB 1	Oversubscribed multiple 1	Number of effective offline subscription shares/number of offline allotment
	OVERSUB 2	Oversubscribed multiple 2	Total number of effective subscriptions/corresponding allotment quantity
	ΔPE	Price–earnings ratio difference	The difference between the P/E ratio of issuance and the industry P/E ratio of reference China Securities Regulatory Commission (CSRC)
Main control variables	SIZE	Company size	Net asset value per share
	EPS	Profitability level	Earnings per share before issuance
	BOARD	Listing board	Main board = 1, medium and small board = 2, GEM = 3

*GEM, growth enterprise market.*

**TABLE 2:** Descriptive statistical analysis results of each variable.

Abbreviation	Mean	Standard deviation	Minimum	Maximum
CAR	3.4016	2.8371	0.1334	18.2362
MR	0.0021	0.0025	−0.0079	0.0010
LOTTERY	0.0050	0.0039	0.0002	0.0288
OVERSUB 1	912.35	1,056.13	33.2447	5,934.26
OVERSUB 2	1,302.41	1,630.23	25.7321	6,955.33
ΔPE	26.7821	22. 2013	−13.72	187.45
SIZE	8.8821	0.3992	7.5326	12.0015
EPS	0.7955	0.4420	0.0698	3.01
BOARD	1.9385	0.9148	1	3

A multiple-linear regression model of IPO abnormal initial returns is established, which can be expressed as shown below.

(1) $$C\,R\,A=\beta_{0}+\beta_{1}\,M\,R+\beta_{2}\,L\,O\,T\,T\,E\,R\,Y+\beta_{3}\,O\,V\,E\,R\,S\,U\,B+\beta_{4}\,\Delta \,P\,E+\beta_{5}\,S\,I\,Z\,E+\beta_{6}\,E\,P\,S+\beta_{7}\,B\,O\,A\,R\,D+\varepsilon$$

#### Source of Sample Data

The sample selected for this study is the data of 522 new shares from 2016 to 2018. The samples used in Model 1 and Model 2 are new stock data from the beginning of 2016 to the end of 2018. The sample used in Model 3 is the new stock data from June 2016 to the end of 2018 because there has been a continuous daily limit for large-scale new stock listings after June 2016.

## Results

### Correlation Test Results of Model Variables

The data type used in this study is cross-section data. The preliminary test is needed to avoid the multiple collinearity between explanatory variables and determine the appropriate variables. The results of the correlation analysis between the explanatory variables and the control variables used in the model are shown in Table 3.

**TABLE 3:** Correlation test results between explanatory variables.

	MR	LOTTERY	OVERSUB 1	OVERSUB 2	ΔPE
MR	1.0000				
LOTTERY	0.0449	1.0000			
OVERSUB 1	−0.1324	−0.4991	1.0000		
OVERSUB 2	−0.1375	−0.5710*	0.9193	1.0000	
ΔPE	0.0021	−0.2913	0.0605	0.06597	1.0000

According to Tables 3, 4, the correlation coefficients between the “oversubscribed multiple 1” in the explanatory variables and the “net asset value per share” in the control variables with other variables are larger. Therefore, these two indicators are selected and added to the model in the empirical process. There is no obvious correlation between “market investor sentiment” and other variables. Then, it can also be added to the model to observe the explanatory effect.

**TABLE 4:** Correlation test results between control variables.

	SIZE	EPS	BOARD
SIZE	1.0000		
EPS	0.8469*	1.0000	
BOARD	0.2162	0.2210	1.0000

### Results of Empirical Analysis

**FIGURE 2:**
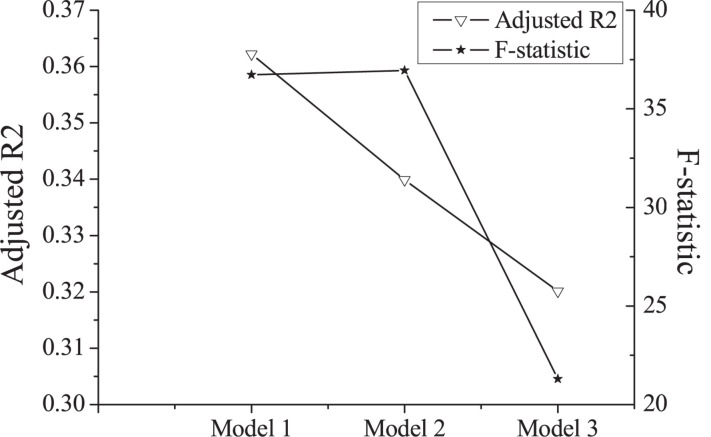
Empirical analysis results.

In the empirical analysis stage, “market investor sentiment” is added to the three models mentioned above. The results obtained are shown in Table 5 and Figure 2. The results obtained are achieved by the least squares regression. The heteroscedasticity-robust standard error is added to the regression process, and the biased error of the model results caused by heteroskedasticity is corrected. As can be seen in Table 5, MR passes the test in all three models, and its significance level is 99%.

**TABLE 5:** Results of empirical analysis.

	Model 1	Model 2	Model 3
Observations	522	522	522
Adjusted *R*^2^	0.3622	0.3399	0.3201
*F*-statistic	36.72	36.94	21.30
Prob	0	0	0

According to a comprehensive analysis, the company size and profitability level in the control variables are negatively correlated with the IPO abnormal initial returns. IPO abnormal initial returns on the main board, SMEs, and GEM have gradually increased. And different listing boards have a significant influence on the IPO excess returns.

### Relationship Between the Initial Public Offering Initial Return and Investor Disagreement

**FIGURE 3:**
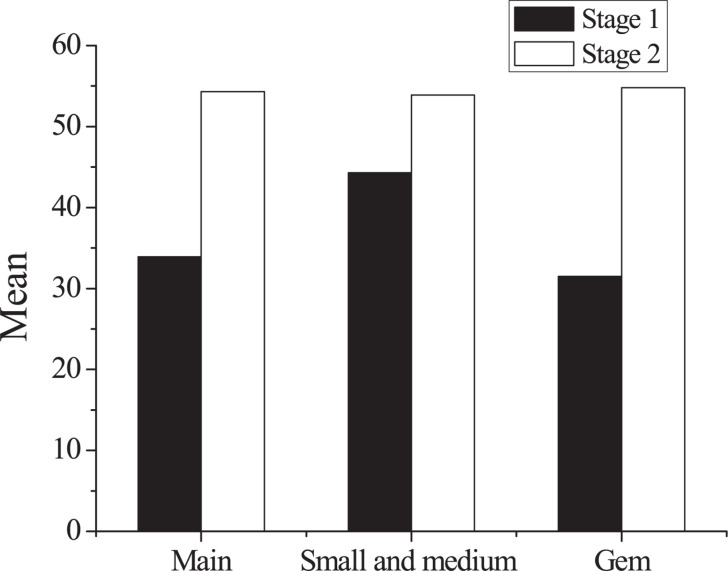
Comparison of average initial public offering underpricing in various boards. GEM, growth enterprise market.

**FIGURE 4:**
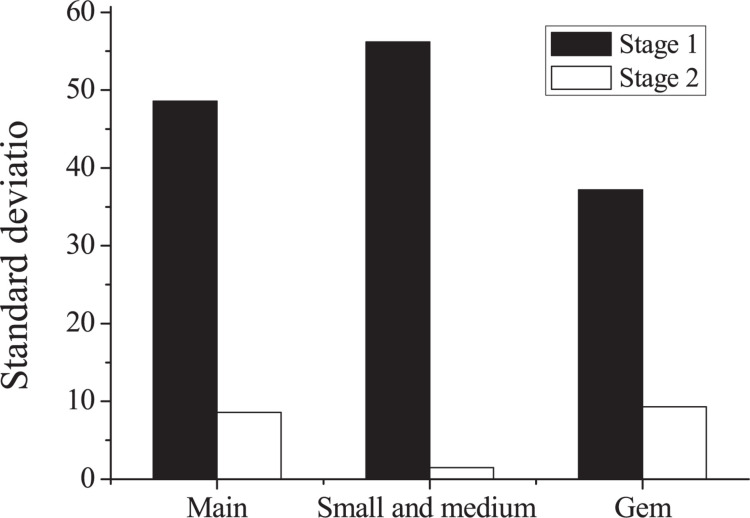
Comparison of standard deviations of initial public offering underpricing in various boards. GEM, growth enterprise market.

This paper makes statistics on the IPO initial returns of the main board, SMEs, and GEM. Descriptive statistics on IPO underpricing of each board are shown in Table 6 and Figures 3, 4, respectively. According to Table 6, the underpricing rate of the second-stage IPO has significantly improved as compared with the first stage, which is correlated to the overall quality improvement of listed companies and the lower threshold for new stock purchases. It shows that the pricing power of China’s current A-share market has improved. From the perspective of the market board, companies listed in the main board market are large-scale, highly transparent, and have relatively stable performance. Therefore, the IPO underpricing rate in the main board market is relatively low. In the early stages, the IPO initial return of the GEM is not high, which is mainly related to the small size, the short establishment time, and the sluggish market performance of the GEM.

**TABLE 6:** Statistics of initial public offering underpricing in various boards.

Stage	Board	Initial return	
		
		Mean	Standard deviation
Stage 1 (2016∼2017)	Main board	33.9	48.6
	Small and medium board	44.3	56.2
	GEM	31.5	37.2
Stage 2 (2017∼2018)	Main board	54.3	8.6
	Small and medium board	53.9	1.5
	GEM	54.8	9.3

**FIGURE 5:**
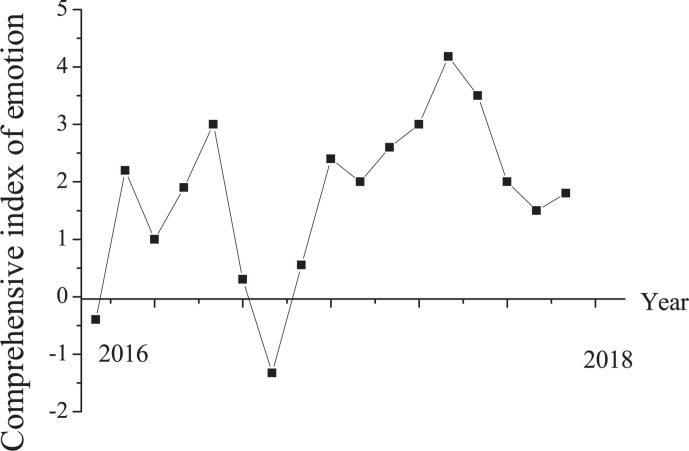
Performance of investor sentiment indicator from 2016 to 2018.

The comprehensive index of investor sentiment was constructed using principal component analysis and sentiment indicators. Through the principal component analysis method, the original variables are recombined into a new set of several unrelated comprehensive variables. At the same time, according to the actual needs, several comprehensive variables can be taken out to reflect the information of the original variables as much as possible. The performance of investors’ comprehensive sentiment indicators from 2016 to 2018 is shown in Figure 5. The average value of investor sentiment indicators is 0, and the standard deviation is 0.33. The minimum and maximum values are −1.33 and 4.18, respectively. It concludes that the sentiment of Chinese investors is relatively stable during the sampling period.

For GEM listed companies, the higher the return on the GEM, the smaller the issue size and the lower the issue price. Companies with higher profitability have higher IPO returns. Whether it is the primary market or the secondary market, the higher the investor sentiment, the higher the GEM IPO return will be.

From the perspective of investor sentiment, the excessive unmet demand of investors for new stocks can be analyzed from demand and supply. On the one hand, the concept of undefeated new stocks has gained popularity. Investors are suspected of blind speculation in new stocks. They believe that buying new stocks is equivalent to obtaining income guarantees. As a result, investors tend to rush to most new stocks. Then, the market has excessive demand for new shares. The continuous daily limit of new stock listings has resulted in abnormally high short-term excess returns of new stocks, which has further strengthened the investment enthusiasm of some investors. Also, this kind of behavior has intensified and further promoted the continuous daily limit of new stock listings. On the other hand, China’s new stock issuance is subject to an approval system. Although a certain degree of marketization has been achieved, there are still artificial policies and regulations on the issuance of new shares, which has resulted in the failure of new shares to follow the market’s wishes.

## Discussion

The phenomenon of IPO excess returns in China has been in the market for a long time. Constrained by the initial rise and fall of listing, there has been a continuous daily limit after the listing of new shares. In addition, the continuous record of the number of new shares in the market is constantly being broken, which has made investors eager and optimistic about the prospects of enterprise development. On the surface, the development momentum of the enterprise is sufficient, and the financing effect is good. However, in the short term, the IPO excess return is an unhealthy abnormal market development momentum. In this paper, from the perspective of investor sentiment, investor sentiment is an individual belief formed by investors’ analysis of the market (Yim et al., 2018). Obviously, this belief cannot represent the fair value of the market, but it will affect investors’ decision-making and market behavior activities. From a behavioral finance perspective, investors with heterogeneous beliefs and preferences are often irrational (Zheng et al., 2020). In their decision-making and investment process, psychological factors play a very important role. The large fluctuations in investor sentiment will bring about deviations in market perception, which will cause the stock price to be affected by investor sentiment to a certain extent and change trends. Research shows that investor sentiment has a significant positive effect on the over-raising of new shares. In other words, when investor sentiment is high, the issuing company can obtain more excess funds during this period. The conclusion of the research is almost consistent with the previous scholars’ conclusions. The turnover rate on the first day of listing and the price fluctuation rate on the first day are positively correlated with IPO underpricing. The success rate of online issuance, issuance scale, and issuance price is negatively correlated with IPO underpricing.

In the field of IPO in the stock exchange market, the initial return of listing is an important indicator used to measure IPO underpricing, and it is used as a standard to measure the level of pricing underpricing of new shares in the primary market. In 2014, after China’s new IPO regulations were established, the first-day gain was restricted, which could not reflect the intrinsic value of new shares. Investors’ unmet needs for new shares cannot be effectively released. After the excessive pursuit, after the first day of listing, the stock price is continued to push up to cause the continuous daily limit. The daily limit phenomenon is also a positive signal, which will stimulate investors to further sentimental rise and trigger a daily limit. Eventually, the price of new shares will be severely deviated from the high price of the issue price in the short term, adding pressure to the stable development of the new stock market (Wu and Song, 2019). Through empirical research, this paper analyzes the reasons for the abnormal phenomenon of short-term excess returns of IPO mainly because investor sentiment is not considered in the IPO. At this time, due to policies or inefficiencies in the pricing of new shares, investors have excessive demand for new shares and demand has fallen short of supply. Then, the new stock issuance system in China determines the scarcity of new stock supply (Yan et al., 2019; Feng et al., 2020). Finally, the level of investment of Chinese investors is relatively low, and the overall consideration is insufficient. The phenomenon of irrational behavior exists as a whole.

The irrational factors in China’s new stock market are inseparable from the structural framework of investors. At present, the majority of retail investors among stock market investors still exist. Since they are limited by professional knowledge, it is easy to follow the trend blindly (Heradio et al., 2016). To reduce the blind pursuit of new stocks by investors, this paper recommends that investors should implement value investment and comprehensively evaluate the basic information of newly listed companies. The supervisory authority should pay attention to the influence of investor sentiment and strengthen the timely disclosure of fundamental information of stock issuers. On the premise of protecting the interests of investors, a harmonious situation of investors’ rational participation in the issuance of new shares is constructed (Shen et al., 2019; Shen and Ho, 2020).

## Conclusion

To deal with the abnormal phenomenon that affects the healthy development of the stock market during the IPO issuance and operation, this paper considers the factor of investor sentiment. Based on the information asymmetry theory and behavioral finance perspective, under the influence of investor sentiment, it is clear that the reason for IPO abnormal initial returns is related to the large fluctuation of investor sentiment. Sentiment fluctuations will cause investors to deviate from market perceptions, which will cause the stock price to present a situation of excess returns to a certain extent. Investor sentiment is one of the important drivers of short-term IPO excess returns. It also shows to a certain extent that there are still many irrational factors in China’s new stock market. Unmet demand for new stock purchases will continue to be the main factor in the short-term listing of new stocks, driving the price of new stocks to rise in the secondary market.

In this regard, investors should comprehensively weigh the IPO companies to avoid blind compliance. As the regulatory review department of securities transactions, it should regulate the methods and scope of information disclosure, make the information as transparent as possible, to protect investors’ rights and interests (Xu and Du, 2018). Due to time constraints, the investigation on the long-term performance of the IPO is not involved. Therefore, the impact of investor sentiment on the long-term performance of the IPO can be used as a direction for further investigation.

## Data Availability Statement

The raw data supporting the conclusions of this article will be made available by the authors, without undue reservation.

## Ethics Statement

The studies involving human participants were reviewed and approved by East China Normal University Ethics Committee. The patients/participants provided their written informed consent to participate in this study.

## Author Contributions

All authors listed have made a substantial, direct and intellectual contribution to the work, and approved it for publication.

## Conflict of Interest

The authors declare that the research was conducted in the absence of any commercial or financial relationships that could be construed as a potential conflict of interest.
